# *TRAF1-C5* Affects Quality of Life in Patients with Primary Biliary Cirrhosis

**DOI:** 10.1155/2013/510547

**Published:** 2013-04-28

**Authors:** Joanna Raszeja-Wyszomirska, Ewa Wunsch, Agnieszka Kempinska-Podhorodecka, Daniel S. Smyk, Dimitrios P. Bogdanos, Malgorzata Milkiewicz, Piotr Milkiewicz

**Affiliations:** ^1^Liver Research Laboratories, Pomeranian Medical University, Aleja Powstańców Wielkopolskich 72, 70-111 Szczecin, Poland; ^2^Liver Unit, Department of General Surgery and Liver Transplantation, Warsaw Medical University, ul. Banacha 1a, 02-097 Warsaw, Poland; ^3^Medical Biology Laboratory, Pomeranian Medical University, Aleja Powstańców Wielkopolskich 72 70-111 Szczecin, Poland; ^4^Institute of Liver Studies, King's College London School of Medicine, King's College Hospital, Denmark Hill, London SE5 9RS, UK

## Abstract

*Background*. Previous studies reported associations between specific alleles of non-HLA immunoregulatory genes and higher fatigue scores in patients with primary biliary cirrhosis (PBC). *Aim*. To study the relationship between variables of health-related quality of life (HRQoL) and single nucleotide polymorphisms of *TRAF1-C5*, a member of the tumor necrosis factor receptor family. *Patients and Methods*. *TRAF1-C5* gene polymorphisms, rs2900180 and rs3761847, were analysed in 120 Caucasian PBCs. The HRQoL was assessed with SF-36, PBC-40, and PBC-27 questionnaires. *Results*. We found a negative association between TT genotype of rs2900180 and SF-36's domains vitality (*P* < 0.05), mental health (*P* < 0.05), and mental component summary score (*P* < 0.05). GG homozygotes of rs3761847 had lower vitality (*P* < 0.05), mental health (*P* < 0.05), mental component summary score (*P* < 0.05) and impairment of social functioning (*P* < 0.01). Allelic analysis has shown that T allele of rs2900180 and G allele of rs3761847 related to SF-36's vitality (*P* < 0.05 and *P* < 0.01), social functioning (*P* < 0.05 and *P* < 0.05), mental health (*P* < 0.01 and *P* < 0.05), and mental component summary score (*P* < 0.01 and *P* < 0.05), respectively. Genotyping and allelic analysis did not reveal correlation with PBC-40 and PBC-27 domains. *Conclusion*. The association between rs2900180 and rs3761847 polymorphisms and HRQoL variables indicates that TRAF1 is involved in the induction of impaired QoL in PBC.

## 1. Introduction

Primary biliary cirrhosis (PBC) is a slowly progressive, chronic liver disease characterized histologically by nonsuppurative destruction of intrahepatic bile duct epithelial cells and serologically by the presence of disease-specific antimitochondrial antibodies (AMAs) and antinuclear antibodies (ANA), targeting the sp100 and gp210 antigens [1–3]. Patients with PBC may suffer from various symptoms which can significantly impair health-related quality of life (HRQoL) [4], including fatigue, pruritus, sleep disturbances, cognitive dysfunction, and mood disorders [5].

The aetiology of PBC is poorly understood [6], but environmental and genetic influences have been considered important for the pathogenesis of the disease [4, 5]. Previous genetic studies and recent genome wide association studies (GWAS) have identified a number of non-HLA susceptibility genes and emphasized the pathogenetic role of interleukin (IL)-12 and tumor necrosis factor (TNF) [7–14]. However, very few of those studies associated gene polymorphisms with clinical and immunological features [15, 16]. Donaldson et al. [17] were unable to identify single nucleotide polymorphisms (SNPs) within cytotoxic T lymphocyte antigen-4 (*CTLA4*) as risk factors for PBC and also failed to find significant associations with clinical and histological features of the disease. However, these authors reported higher fatigue scores using the PBC-40 questionnaire in those PBC patients with the *CTLA4* −319 T allele and the *CTLA4* +49 AA genotype, suggesting that these alleles may be linked to the induction of fatigue rather than susceptibility to disease [17]. Akin to these reports, we have recently reported that rs2900180 and rs3761847 SNPs of *TRAF1-C5* are similarly prevalent in patients with PBC and in healthy controls but are associated with a more frequent presence of PBC-specific gp210 autoantibodies [18]. Our data suggest that *TRAF1-C5*, an autoimmune disease risk locus, may participate in the loss of immunological tolerance to this nuclear complex protein in PBC [18]. 

TNF receptor-associated factor-1 (TRAF1) and Complement 5 (C5) (*TRAF1-C5*) lie adjacent to one another on 9q33-34 chromosome and confer susceptibility to diseases such as rheumatoid arthritis (RA) and systemic lupus erythematosus (SLE) [19–22]. *TRAF1* is associated with multiple TNF-receptor family members and controls cytokine signaling cross-talking, including that of TNF*α* through the binding of adaptor proteins and protein kinases [23], while C5 dysregulation leads to disorganized complement activation and overt autoimmune disease, including PBC [24–29].

In the present study, we have sought to assess whether the presence of rs2900180 and rs3761847 TRAF1 SNPs is associated with features related to HRQoL in patients with PBC. Our working hypothesis linking *TRAF1-C5* with HRQoL features is based on experimental work performed in animal models, which have shown that chronic cholestasis-associated sickness behaviours may relate to enhanced interleukin 1 *β* expression and recruitment of TNF*α*-producing monocytes into the brain, possibly as the result of the activation of cerebral endothelium [30, 31]. TNF*α* increase associates with malaise and mood disorders in patients with chronic fatigue syndrome, clearly underlying the important pathogenic role of this proinflammatory cytokine [32–34]. *TRAF1* regulates CD40 and TNF transduction signaling and may play a role in cholestasis-induced sick behaviours mediated by TNF*α* [30–36]. To this end, we have analyzed features of HRQoL and their association with *TRAF1-C5* polymorphisms in a well-defined cohort of PBC patients.

## 2. Patients and Methods

### 2.1. Study Group

This is a retrospective analysis of prospectively collected data that includes a homogenous cohort of 120 Polish patients with PBC consecutively seen in the Liver Unit and the Liver Out-Patient Clinic of our institution [18]. All patients met the criteria for the diagnosis of PBC according to the latest guidelines issued by the European Association for the Study of the Liver [37]. The clinical and laboratory characteristics of the patients are summarized in Table 1. Overall, 33 (28%) patients had either histological or clinical/imaging evidence of liver cirrhosis. A status of fibrosis has not been established in 2 patients with PBC. 

### 2.2. HRQoL Symptoms Assessment

The HRQoL was assessed with both generic (the Medical Outcomes Study Short Form-36, SF-36) and disease-specific questionnaires (PBC-40 and PBC-27). The SF-36 is a widely used and validated generic HRQoL questionnaire, which includes 36 items divided into 8 scales. Scores can be obtained for each scale or can be aggregated into 2 summary scores, a *mental component summary* score and a *physical component summary* score. Scale scores range between 0 and 100, with the higher score indicating better HRQoL [38].

PBC-40 is a disease-specific 40-item HRQoL questionnaire, designed for self-completion in patients with PBC. It consists of specific symptom domains *(cognition, itch, fatigue, social and emotional, and other symptoms)*, marked with a 5-point scale (1 = never to 5 = always), with higher scores denoting greater symptoms impact and poorer quality of life [39]. PBC-27 is an alternative PBC-specific HRQoL questionnaire, developed as a simplification of the PBC-40 form. PBC-27 was shown to be equally effective in detecting the impact of PBC on HRQoL [40]. It contains 27 items grouped in 7 domains: *other symptoms, dryness, itch, fatigue, cognitive, emotional*, and *social* with the same 5-point scale of evaluation.

### 2.3. *TRAF1-C5* Genotyping

Two *TRAF1-C5* gene SNPs, rs2900180 and rs3761847, were analyzed, as previously described [18]. Briefly, DNA from peripheral blood mononuclear cells was isolated using the DNeasy Blood & Tissue Kit (Qiagen) [18]. Oligonucleotide primers and TaqMan probes for the *TRAF1* polymorphisms were designed and synthesized by Applied Biosystems (Assay ID: C_15849116_10 and C_2783640_10, resp.) [18]. The fluorescence data were analyzed with allelic discrimination 7500 Software v.2.0.2.

### 2.4. Ethics

Appropriate informed consent was obtained from each patient included in the study. The study protocol was approved by the Ethics Committee of Pomeranian Medical University and conformed to the Ethical Guidelines of the 1975 Declaration of Helsinki (6th revision, 2008).

### 2.5. Statistical Analysis

Data are shown as means and standard deviations. All statistical analyses were carried out using StatView software (Carry, NC, US). The genotype and allelic frequencies were compared using Fisher's PLSD test. The analysis of genotype frequency in relation to HRQoL was performed using Anova. *P* values less than 0.05 were considered to be statistically significant.

## 3. Results

Significant negative associations between TT genotype of rs2900180 and 3 domains of SF-36 were found. These included vitality (*P* < 0.05), mental health (*P* < 0.05), and mental component summary score (*P* < 0.05). Also, subjects who were GG homozygotes of the rs3761847 had a significant impairment of social functioning (*P* < 0.01), significantly lower vitality (*P* < 0.05), mental health (*P* < 0.05), and mental component summary score (*P* < 0.05). These data are summarised in Table 2. 

No correlation was found between genotypes of both analyzed SNPs and any domains of PBC-40 and PBC-27 questionnaires. These data are shown in Tables 3 and 4.

Allelic analysis demonstrated that T allele of rs2900180 and G allele of rs3761847 related to impaired mental health in several SF-36 items, including vitality (*P* < 0.05 and *P* < 0.01), social functioning (*P* < 0.05 and *P* < 0.05), mental health (*P* < 0.01 and *P* < 0.05), and mental component summary score (*P* < 0.01 and *P* < 0.05), respectively. These data are summarized in Table 5. 

No correlation was found between rs2900180 and rs3761847 gene polymorphisms and quality of life features using the PBC-40 and PBC-27 questionnaires. These data are shown in Tables 6 and 7. 

These results were independent of the stage of fibrosis, as the proportion of patients with liver cirrhosis was comparable amongst the groups under analysis. In TT homozygotes of rs2900180 there were 3 (23%) subjects with cirrhosis, as compared to 15 (29%) in AA homozygotes group (*P* = 0.87). Similarly in GG homozygotes of the rs3761847, there were 7 (30%) patients with cirrhosis as compared to 13 (32%) in CC homozygotes group (*P* = 0.81).

## 4. Discussion

In the present study we evaluated the association between *TRAF1-C5* rs2900180 and rs3761847 polymorphisms and variables of HRQoL in a homogenous group of Caucasian patients with PBC [18]. Using both generic and disease-specific HRQoL tools, we have been able to identify a clear relationship between the presence of the T allele of rs2900180 and the G allele of rs3761847 of *TRAF1-C5* with impaired mental HRQoL, as measured by the SF-36 questionnaire. Our study shows that TRAF1-C5 may have clinical relevance for the identification of PBC patients with features of impaired well-being. *TRAF1-C5* polymorphisms confer susceptibility to RA and SLE [20, 21, 41–46] and have been shown to be prognosticators of the natural course of the disease [47–49]. 

In our previous study, we found that rs2900180 and rs3761847 are similarly present in PBC and controls [18]. In that study, we were unable to identify any clinically meaningful association related to the presence of these polymorphisms, with the exception of an association with anti-gp210 antibody [18], a prognostically relevant autoantibody marker of PBC [50–52]. Our findings relating *TRAF-C5* polymorphisms with HRQoL are unrelated to the stage of fibrosis of the analyzed groups, further underlying the significance of the genetic associations with the features of impaired HRQoL. The exact role played by *TRAF1-C5* and its strict association with sick behaviours is far from clear. *TRAF1-C5* plays an important role in the homeostasis of TNF-*α*, as well as a plethora of other cytokines with important pro-inflammatory actions [23, 35, 36]. Cholestatic disorders such as PBC are characterised by an imbalance of circulating pro-inflammatory and anti-inflammatory cytokines. This dysregulation may directly or indirectly influence sick behaviours, as the enhanced release of TNF-*α* within the brain contributes to the development of mood disorders and mental sickness [30–36]. In an animal model of cholestasis, Kerfoot et al. [31] have demonstrated in an increased infiltration of monocytes producing TNF*α*, which subsequently leads to further activation of resident brain macrophages able to produce TNF*α*. Also, a fine Th1/Th2 immunity imbalance affects the IL-10/IL-12 regulatory circuit [14, 53, 54] and leads to over expression of IL-12 and TNF-*α* cytokines in patients with chronic fatigue. An increase of circulating TNF-*α* levels has been associated with significant fatigue in cholestatic patients with cancer [55]. Finally, work on animal models of chronic fatigue syndrome has also underlined the important pathogenic role of TRAF1-related cytokines, such as TNF*α* [32, 34]. These findings warrant further investigation. 

Several studies have shown that PBC is associated with impaired HRQoL. Poupon et al. [56] described a significant impairment of energy and emotional reactions in patients with PBC. Younossi et al. [57] found that in patients with PBC, *physical component summary* of SF-36 scores weas decreased, with a further decline as disease progressed. No other demographic and clinical parameters were associated with HRQoL. In that study, *bodily pain*, *mental health *and *social functioning* domains of SF-36 reached the highest scores, and *physical component summary* was lower than *mental component summary* score in PBC [57]. Using the SF-36 questionnaire, Wong et al. [58] reported a significantly lower value of *physical component summary* and slightly lower value of *mental component summary* in 44 patients with PBC. Those authors reported that fatigue mainly affects the mental aspects of HRQoL and, to a lesser extent, the physical components [58]. That study also showed significantly lower values of *vitality* and *social functioning *domains of SF-36 [58]. Sogolow et al. [59] studied 80 females with PBC and reported poorer HRQoL for the *physical *rather than for *mental component summary*. As well, the values for* social functioning* and *mental health* domains were significantly decreased in that study. A greater physical impairment of HRQoL in patients with PBC as compared to autoimmune hepatitis and hepatitis C was also reported by Tillmann et al. [60]. The reported data argue for extreme caution in interpreting results in the light of the weaknesses and the discrepancies noted. We have been unable to show any association between analysed polymorphisms and domains of PBC-specific questionnaires such as PBC-40 or PBC-27. These domains have been considered useful tools in the assessment of quality of life in PBC, and one would expect them to show correlations with the *TRAF1-C5* polymorphisms. However, this has not been proven to be the case. We can only speculate that in this particular situation, domains of generic SF-36 may express those aspects of quality of life better. This topic needs external validation in greater numbers of patients.

In conclusion, we have provided evidence in support of the thesis that the genetic makeup of patients with PBC may influence features related to the quality of life associated with immune dysregulation. Whether these associations are epiphenomena or have true meaning in terms of the induction and the course of the disease will be investigated in the years to come.

## Figures and Tables

**Table 1:** Clinical and laboratory features of 120 patients with primary biliary cirrhosis (PBC) participated in the study.

Feature	PBC (*n* = 120)
Age (median; range)	56 (22–83)
Gender (M/F)	11/109
AMA (pos/neg)	101/19
*Anti-sp100 (pos/neg)	27/92
*Anti-gp210 (pos/neg)	15/104
**Cirrhosis (yes/no) (%)	33/85 (28/72%)
ALT (median; range) IU/L (Normal: 3–30)	90 (10–987)
ALP (median; range ), IU/L (Normal: 40–120)	304 (47–2264)
GGT (median; range), IU/L (Normal: 3–30)	311 (11–2608)
Bilirubin (median; range), mg/dL (Normal: 0.2–1.0)	1.9 (0.2–25.1)
Albumin (median; range), g/dL (Normal: 3.8–4.4)	3.9 (2.3–5.8)
INR (median; range) (Normal: 0.8–1.2)	1.04 (0.8–2.3)
Cholesterol (median; range ), mg/dL (Normal < 200)	249 (108–1096)
Triglycerides (median; range), mg/dL (Normal < 150)	124 (42–448)

*In 1 patient these tests were not done; **in 2 patients fibrosis status was not determined.

M: male; F: female; AMA: antimitochondrial antibody; Pos: positive; Neg: negative.

**Table 2:** Relationship between features of SF-36 and analyzed polymorphisms.

SF-36	rs 2900180			rs 3761847		
Domain	CC	TT	*P*	AA	GG	*P*
Physical functioning	67.4 ± 3.8	66.8 ± 7.0	*0.95 *	66.7 ± 4.1	61.7 ± 4.7	*0.48 *
Role-physical	44.6 ± 6.2	27.3 ± 11.4	*0.21 *	46.6 ± 6.9	28.6 ± 5.1	*0.12 *
Bodily pain	57.1 ± 3.8	51.2 ± 5.7	*0.49 *	56.0 ± 4.1	48.9 ± 5.1	*0.32 *
General health	48.5 ± 2.9	46.4 ± 4.3	*0.76 *	46.7 ± 3.1	45.4 ± 3.7	*0.82 *
Vitality	56.1 ± 3.3	40.8 ± 5.2	**<0.05**	56.8 ± 3.7	41.6 ± 3.5	**<0.05**
Social functioning	71.7 ± 3.7	55.7 ± 5.9	*0.05 *	72.9 ± 3.9	54.2 ± 4.2	**<0.01**
Role emotional	55.1 ± 6.7	36.4 ± 10.5	*0.22 *	59.5 ± 7.1	35.0 ± 8.5	*0.05 *
Mental health	66.9 ± 2.9	48.4 ± 5.1	**<0.05**	67.5 ± 3.3	55.2 ± 3.9	**<0.05**
Physical component summary	42.9 ± 1.4	43.2 ± 2.6	*0.93 *	41.7 ± 1.4	40.9 ± 1.9	*0.78 *
Mental component summary	46.5 ± 1.9	36.4 ± 1.9	**<0.05**	46.9 ± 1.913	38.2 ± 1.7	**<0.05**

**Table 3:** Relationship between rs2900180 and rs3761847 *TRAF1-C5* polymorphisms and PBC-40 domains.

PBC-40	rs2900180			rs3761847		
Domain	CC	TT	*P*	AA	GG	*P*
Other symptoms	16.5 ± 0.7	17.3 ± 1.2	*0.61 *	16.5 ± 0.8	17.9 ± 1.0	*0.29 *
Itch	9.9 ± 0.6	5.6 ± 1.1	*0.19 *	4.3 ± 0.7	6.1 ± 0.8	*0.10 *
Fatigue	26.1 ± 1.5	28.8 ± 3.0	*0.39 *	26.4 ± 1.7	29.9 ± 2.1	*0.20 *
Cognitive	12.9 ± 0.7	13.4 ± 1.7	*0.79 *	13.0 ± 0.8	14.1 ± 1.3	*0.46 *
Social and emotional	28.5 ± 2.9	28.5 ± 2.9	*0.94 *	29.6 ± 1.5	29.1 ± 2.1	*0.88 *

**Table 4:** Relationship between rs2900180 and rs3761847 *TRAF1-C5* polymorphisms and PBC-27 questionnaire.

PBC-27	rs2900180			rs3761847		
Domain	CC	TT	*P*	AA	GG	*P*
Other symptoms	7.7 ± 0.4	7.7 ± 0.6	*0.98 *	7.8 ± 0.5	8.5 ± 0.5	*0.38 *
Dryness	5.0 ± 0.3	5.8 ± 0.6	*0.27 *	4.9 ± 0.3	6.0 ± 0.5	*0.07 *
Itch	9.9 ± 0.6	5.6 ± 1.1	*0.19 *	4.3 ± 0.7	6.1 ± 0.8	*0.10 *
Fatigue	19.0 ± 1.0	20.5 ± 2.2	*0.50 *	19.3 ± 1.1	21.9 ± 1.5	*0.24 *
Cognitive	11.1 ± 0.6	11.0 ± 1.4	*0.96 *	11.3 ± 0.7	11.6 ± 1.0	*0.84 *
Social	7.3 ± 0.4	5.9 ± 0.8	*0.18 *	7.5 ± 0.5	6.3 ± 0.7	*0.19 *
Emotional	6.5 ± 0.4	7.5 ± 1.0	*0.34 *	6.8 ± 0.4	7.3 ± 0.8	*0.52 *

**Table 5:** Allelic analysis (C versus T and A versus G) of rs 2900180 and rs 3761847, respectively, in relation to SF-36 domains.

SF-36	rs2900180			rs3761847		
	C	T	*P*	A	G	*P*
Physical functioning	67.4 ± 3.8	60.3 ± 3.4	*0.17 *	66.7 ± 4.1	61.6 ± 3.2	*0.34 *
Role-physical	44.6 ± 6.2	35.7 ± 5.0	*0.26 *	46.6 ± 6.9	35.8 ± 4.7	*0.19 *
Bodily pain	57.1 ± 3.8	49.4 ± 3.3	*0.12 *	56.1 ± 4.1	50.9 ± 3.1	*0.33 *
General health	48.5 ± 2.9	45.3 ± 2.6	*0.42 *	46.7 ± 3.1	46.6 ± 2.4	*0.98 *
Vitality	56.1 ± 3.3	43.2 ± 2.6	**<0.05**	56.8 ± 3.7	44.5 ± 2.5	**<0.01**
Social functioning	71.7 ± 3.7	59.5 ± 3.0	**<0.05**	72.9 ± 3.9	60.4 ± 2.9	**<0.05**
Role emotional	55.1 ± 6.7	44.8 ± 5.8	*0.25 *	59.5 ± 7.1	43.8 ± 5.4	*0.09 *
Mental health	66.9 ± 2.9	55.2 ± 2.8	**<0.01**	67.5 ± 3.3	56.5 ± 2.6	**<0.05**
Physical component summary	42.9 ± 1.4	40.4 ± 1.3	*0.21 *	41.7 ± 1.4	41.3 ± 1.3	*0.84 *
Mental component summary	46.5 ± 1.9	39.8 ± 1.5	**<0.01**	46.9 ± 1.9	40.4 ± 1.5	**<0.05**

**Table 6:** Allelic analysis (C versus T and A versus G) of rs 2900180 and rs 3761847, respectively, in relationship to PBC-40 domains.

PBC-40	rs2900180			rs3761847		
	C	T	*P*	A	G	*P*
Other symptom	16.5 ± 0.7	17.2 ± 0.7	*0.48 *	16.5 ± 0.8	17.0 ± 0.6	*0.61 *
Itch	3.9 ± 0.6	4.8 ± 0.5	*0.24 *	4.3 ± 0.7	4.5 ± 0.5	*0.82 *
Fatigue	26.1 ± 1.5	29.7 ± 1.2	*0.07 *	26.4 ± 1.7	28.9 ± 1.2	*0.21 *
Cognitive	12.9 ± 0.7	13.9 ± 0.7	*0.33 *	13.0 ± 0.8	13.7 ± 0.6	*0.50 *
Social and emotional	28.8 ± 1.5	30.3 ± 1.3	*0.46 *	29.6 ± 1.5	29.6 ± 1.3	*0.97 *

**Table 7:** Allelic analysis (C versus T and A versus G) of rs 2900180 and rs 3761847, respectively, in relation to PBC-27 domains.

PBC-27	rs2900180			rs3761847		
	C	T	*P*	A	G	*P*
Other symptom	7.7 ± 0.4	8.2 ± 0.4	*0.33 *	7.3 ± 0.4	7.5 ± 0.4	*0.78 *
Dryness	5.1 ± 0.3	5.4 ± 0.3	*0.38 *	4.9 ± 0.3	5.4 ± 0.3	*0.24 *
Itch	3.9 ± 0.6	4.8 ± 0.5	*0.24 *	4.3 ± 0.7	4.5 ± 0.5	*0.82 *
Fatigue	19.1 ± 1.1	21.6 ± 0.9	*0.05 *	19.3 ± 1.1	21.1 ± 0.8	*0.19 *
Cognitive	11.1 ± 0.6	11.6 ± 0.6	*0.57 *	11.3 ± 0.7	11.4 ± 0.5	*0.96 *
Emotional	6.5 ± 0.4	7.2 ± 0.4	*0.22 *	6.8 ± 0.4	6.9 ± 0.4	*0.75 *
Social	7.3 ± 0.4	7.5 ± 0.4	*0.78 *	7.5 ± 0.5	7.3 ± 0.4	*0.84 *

## Abbreviations

AMA: Antimitochondrial antibodies
HRQoL: Health-related quality of life
PBC: Primary biliary cirrhosis
RA: Rheumatoid arthritis
SLE: Systemic lupus erythematous
SNP: Single nucleotide polymorphism
TNF: Tumor necrosis factor
TRAF1: Tumor necrosis factor receptor-associated factor-1.

## References

[1] Kaplan MM, Gershwin ME (2005). Primary biliary cirrhosis. *The New England Journal of Medicine*.

[2] Bogdanos DP, Komorowski L (2011). Disease-specific autoantibodies in primary biliary cirrhosis. *Clinica Chimica Acta*.

[3] Bogdanos DP, Invernizzi P, Mackay IR, Vergani D (2008). Autoimmune liver serology: current diagnostic and clinical challenges. *World Journal of Gastroenterology*.

[4] Bogdanos DP, Smyk DS, Rigopoulou EI (2012). Twin studies in autoimmune disease: genetics, gender and environment. *Journal of Autoimmunity*.

[5] Selmi C, Gershwin ME (2009). The role of environmental factors in primary biliary cirrhosis. *Trends in Immunology*.

[6] Gershwin ME, Mackay IR (2008). The causes of primary biliary cirrhosis: convenient and inconvenient truths. *Hepatology*.

[7] Hirschfield GM, Liu X, Xu C (2009). Primary biliary cirrhosis associated with HLA, IL12A, and IL12RB2 variants. *The New England Journal of Medicine*.

[8] Hirschfield GM, Liu X, Han Y (2010). Variants at IRF5-TNPO3, 17q12-21 and MMEL1 are associated with primary biliary cirrhosis. *Nature Genetics*.

[9] Liu X, Invernizzi P, Lu Y (2010). Genome-wide meta-analyses identify three loci associated with primary biliary cirrhosis. *Nature Genetics*.

[10] Mells GF, Floyd JAB, Morley KI (2011). Genome-wide association study identifies 12 new susceptibility loci for primary biliary cirrhosis. *Nature Genetics*.

[11] Juran BD, Hirschfield GM, Invernizzi P (2012). Immunochip analyses identify a novel risk locus for primary biliary cirrhosis at 13q14, multiple independent associations at four established risk loci and epistasis between 1p31 and 7q32 risk Variants. *Human Molecular Genetics*.

[12] Liu JZ, Almarri MA, Gaffney DJ (2012). Dense fine-mapping study identifies new susceptibility loci for primary biliary cirrhosis. *Nature Genetics*.

[13] Nakamura M, Nishida N, Kawashima M (2012). Genome-wide association study identifies TNFSF15 and POU2AF1 as susceptibility loci for primary biliary cirrhosis in the Japanese population. *The American Journal of Human Genetics*.

[14] Lleo A, Gershwin ME, Mantovani A, Invernizzi P (2012). Towards common denominators in primary biliary cirrhosis: the role of IL-12. *Journal of Hepatology*.

[15] Tanaka A, Quaranta S, Mattalia A (1999). The tumor necrosis factor-*α* promoter correlates with progression of primary biliary cirrhosis. *Journal of Hepatology*.

[16] Jones DEJ, Watt FE, Grove J (1999). Tumour necrosis factor-*α* promoter polymorphisms in primary biliary cirrhosis. *Journal of Hepatology*.

[17] Donaldson P, Veeramani S, Baragiotta A (2007). Cytotoxic T-lymphocyte-associated antigen-4 single nucleotide polymorphisms and haplotypes in primary biliary cirrhosis. *Clinical Gastroenterology and Hepatology*.

[18] Kempinska-Podhorodecka A, Shums Z, Wunsch E (2012). TRAF1 gene polymorphism correlates with the titre of Gp210 antibody in patients with primary biliary cirrhosis. *Clinical and Developmental Immunology*.

[19] Kurreeman FAS, Rocha D, Houwing-Duistermaat J (2008). Replication of the tumor necrosis factor receptor-associated factor 1/complement component 5 region as a susceptibility locus for rheumatoid arthritis in a european family-based study. *Arthritis and Rheumatism*.

[20] Zervou MI, Sidiropoulos P, Petraki E (2008). Association of a TRAF1 and a STAT4 gene polymorphism with increased risk for rheumatoid arthritis in a genetically homogeneous population. *Human Immunology*.

[21] Plenge RM, Seielstad M, Padyukov L (2007). TRAF1-C5 as a risk locus for rheumatoid arthritis: a genomewide study. *The New England Journal of Medicine*.

[22] Kurreeman FAS, Goulielmos GN, Alizadeh BZ (2010). The TRAF1-C5 region on chromosome 9q33 is associated with multiple autoimmune diseases. *Annals of the Rheumatic Diseases*.

[23] Lee SY, Choi Y (2007). TRAF1 and its biological functions. *Advances in Experimental Medicine and Biology*.

[24] Bao L, Quigg RJ (2007). Complement in Lupus Nephritis: the good, the bad, and the unknown. *Seminars in Nephrology*.

[25] Sacks SH (2010). Complement fragments C3a and C5a: the salt and pepper of the immune response. *European Journal of Immunology*.

[26] Atkinson JP (2006). C5a and Fc*γ* receptors: a mutual admiration society. *Journal of Clinical Investigation*.

[27] Schmidt RE, Gessner JE (2005). Fc receptors and their interaction with complement in autoimmunity. *Immunology Letters*.

[28] Barak V, Selmi C, Schlesinger M (2009). Serum inflammatory cytokines, complement components, and soluble interleukin 2 receptor in primary biliary cirrhosis. *Journal of Autoimmunity*.

[29] Schlesinger M, Benbassat C, Shoenfeld Y (1992). Complement profile in primary biliary cirrhosis. *Immunologic Research*.

[30] Swain MG, Beck P, Rioux K, Le T (1998). Augmented interleukin-1*β*-induced depression of locomotor activity in cholestatic rats. *Hepatology*.

[31] Kerfoot SM, D’Mello C, Nguyen H (2006). TNF-*α*-secreting monocytes are recruited into the brain of cholestatic mice. *Hepatology*.

[32] Maes M, Twisk FN, Ringel K (2012). Inflammatory and cell-mediated immune biomarkers in myalgic encephalomyelitis/chronic fatigue syndrome and depression: inflammatory markers are higher in myalgic encephalomyelitis/chronic fatigue syndrome than in depression. *Psychotherapy and Psychosomatics*.

[33] Hermann GE, Rogers RC (2008). TNF*α*: a trigger of autonomic dysfunction. *Neuroscientist*.

[34] Jiang Y, Deacon R, Anthony DC, Campbell SJ (2008). Inhibition of peripheral TNF can block the malaise associated with CNS inflammatory diseases. *Neurobiology of Disease*.

[35] Fotin-Mleczek M, Henkler F, Hausser A (2004). Tumor Necrosis Factor Receptor-associated Factor (TRAF) 1 Regulates CD40-induced TRAF2-mediated NF-*κ*B Activation. *Journal of Biological Chemistry*.

[36] Xie P, Hostager BS, Munroe ME, Moore CR, Bishop GA (2006). Cooperation between TNF receptor-associated factors 1 and 2 in CD40 signaling. *Journal of Immunology*.

[37] European Association for the Study of the Liver (2009). EASL Clinical Practice Guidelines: management of cholestatic liver diseases. *Journal of Hepatology*.

[38] Ware JE, Sherbourne CD (1992). The MOS 36-item short-form health survey (SF-36). I. Conceptual framework and item selection. *Medical Care*.

[39] Jacoby A, Rannard A, Buck D (2005). Development, validation, and evaluation of the PBC-40, a disease specific health related quality of life measure for primary biliary cirrhosis. *Gut*.

[40] Montali L, Tanaka A, Riva P (2010). A short version of a HRQoL questionnaire for Italian and Japanese patients with Primary Biliary Cirrhosis. *Digestive and Liver Disease*.

[41] Zhu J, Zhang D, Wu F (2011). Single nucleotide polymorphisms at the TRAF1/C5 locus are associated with rheumatoid arthritis in a Han Chinese population. *BMC Medical Genetics*.

[42] Redler S, Brockschmidt FF, Forstbauer L (2010). The TRAF1/C5 locus confers risk for familial and severe alopecia areata. *British Journal of Dermatology*.

[43] Potter C, Eyre S, Cope A, Worthington J, Barton A (2007). Investigation of association between the TRAF family genes and RA susceptibility. *Annals of the Rheumatic Diseases*.

[44] Nishimoto K, Kochi Y, Ikari K (2010). Association study of TRAF1-C5 polymorphisms with susceptibility to rheumatoid arthritis and systemic lupus erythematosus in Japanese. *Annals of the Rheumatic Diseases*.

[45] Hughes LB, Reynolds RJ, Brown EE (2010). Most common single-nucleotide polymorphisms associated with rheumatoid arthritis in persons of European ancestry confer risk of rheumatoid arthritis in African Americans. *Arthritis Care and Research*.

[46] Han TU, Bang SY, Kang C, Bae SC (2009). TRAF1 polymorphisms associated with rheumatoid arthritis susceptibility in asians and in caucasians. *Arthritis and Rheumatism*.

[47] Knevel R, de Rooy DP, Gregersen PK, Lindqvist E (2012). Studying associations between variants in TRAF1-C5 and TNFAIP3-OLIG3 and the progression of joint destruction in rheumatoid arthritis in multiple cohorts. *Annals of the Rheumatic Diseases*.

[48] Mohamed RH, Pasha HF, El-Shahawy EE (2012). Influence of TRAF1/C5 and STAT4 genes polymorphisms on susceptibility and severity of rheumatoid arthritis in Egyptian population. *Cellular Immunology*.

[49] Morgan AW, Robinson JI, Conaghan PG (2010). Evaluation of the rheumatoid arthritis susceptibility loci HLA-DRB1, PTPN22, OLIG3/TNFAIP3, STAT4 and TRAF1/C5 in an inception cohort. *Arthritis Research and Therapy*.

[50] Wesierska-Gadek J, Penner E, Battezzati PM (2006). Correlation of initial autoantibody profile and clinical outcome in primary biliary cirrhosis. *Hepatology*.

[51] Nakamura M, Kondo H, Mori T (2007). Anti-gp210 and anti-centromere antibodies are different risk factors for the progression of primary biliary cirrhosis. *Hepatology*.

[52] Itoh S, Ichida T, Yoshida T (1998). Autoantibodies against a 210 kDa glycoprotein of the nuclear pore complex as a prognostic marker in patients with primary biliary cirrhosis. *Journal of Gastroenterology and Hepatology*.

[53] Visser J, Graffelman W, Blauw B (2001). LPS-induced IL-10 production in whole blood cultures from chronic fatigue syndrome patients is increased but supersensitive to inhibition by dexamethasone. *Journal of Neuroimmunology*.

[54] White AT, Light AR, Hughen RW (2010). Severity of symptom flare after moderate exercise is linked to cytokine activity in chronic fatigue syndrome. *Psychophysiology*.

[55] Pavese I, Satta F, Todi F (2009). High serum levels of TNF-*α* and IL-6 predict the clinical outcome of treatment with human recombinant erythropoietin in anaemic cancer patients. *Annals of Oncology*.

[56] Poupon RE, Chrétien Y, Chazouillères O, Poupon R, Chwalow J (2004). Quality of life in patients with primary biliary cirrhosis. *Hepatology*.

[57] Younossi ZM, Kiwi ML, Boparai N, Price LL, Guyatt G (2000). Cholestatic liver diseases and health-related quality of life. *The American Journal of Gastroenterology*.

[58] Wong GLH, Law FMY, Wong VWS (2008). Health-related quality of life in Chinese patients with primary biliary cirrhosis. *Journal of Gastroenterology and Hepatology*.

[59] Sogolow ED, Lasker JN, Short LM (2008). Fatigue as a major predictor of quality of life in women with autoimmune liver disease. The case of primary biliary cirrhosis. *Women’s Health Issues*.

[60] Tillmann HL, Wiese M, Braun Y (2011). Quality of life in patients with various liver diseases: patients with HCV show greater mental impairment, while patients with PBC have greater physical impairment. *Journal of Viral Hepatitis*.

